# Cause of high risk of cervical cancer in socially unclassified women.

**DOI:** 10.1038/bjc.1978.180

**Published:** 1978-07

**Authors:** K. Sibary, R. W. Burslem, J. Wakefield


*Br. J. Cancer* (1978) **38**, 166

***Short Communication***

### CAUSE OF HIGH RISK OF CERVICAL CANCER IN SOCIALLY UNCLASSIFIED WOMEN

K. SIBARY*, R. W. BURSLEM† AND JOHN WAKEFIELD*‡

*From the *Department of Social Research, University Hospital of South Manchester, Christie Hospital and Holt Radium Institute, and the †Department of Obstetrics and Gynaecology, University Hospital of South Manchester, Withington Hospital, Manchester*

Received 14 April 1978 Accepted 26 April 1978

IN A SERIES of studies of a very large cytological screening programme in North-Western England (reviewed by Wakefield, 1972), calculations of the rates of abnormal smears by social class have had to be based on the assumption that women who were excluded from analyses because their records lacked sufficient occupational information would not differ significantly in social-class distribution from those who were classifiable (Wakefield and Sansom, 1966; Sansom *et al.*, 1971). It was clear, however, that these unclassifiable women were in some way uncharacteristic of the larger population of screened women, because they had a high rate of positive and suspicious smears (14·2 per 1000 women, compared with 9·7 for the classifiable) which at first sight suggests that they might include a greater than expected proportion of the high-risk women from Classes IV and V.

Unfortunately, between 10 and 15% of the cytology request forms lacked the information about occupation that is needed to allocate the women to one of the Registrar-General's 6 social classes. As a result, some 71,000 women who had had a smear, being otherwise unclassifiable, were, for the purposes of analyses, allocated to a notional Class VII.

Mortality from carcinoma of the cervix and yields of abnormal smears through cytological screening have been shown in a number of studies, to occur more frequently in women whose husbands have been employed in occupations in the lower range of the Registrar-General's social scale, Classes IV and V (Sansom *et al.*, 1971; Registrar General, 1961; Wakefield *et al.*, 1973). As well as strengthening the view that *in situ* and invasive carcinoma are stages of the same disease, information related to social class has helped to identify high-risk groups and provide a useful way of measuring the extent to which the women most likely to benefit from a cervical screening service have responded to the offer of cytological examination. The exclusion from analyses of one of the groups with the highest abnormal smear rates has, therefore, caused us some anxiety, and placed in question the accuracy of some of our previous findings regarding the social-class gradient in risk of the disease.

The aim of the study reported here was to determine the true class distribution of those women previously unclassified. From that and other information we then hoped to assess the impact of redistribution of the unclassified on the existing social-class gradient in rates of abnormal findings, and to consider the factors responsible for both the high rate of abnormal findings from the previously

‡ Who died tragically and suddenly 10 January 1978

unclassified women and the lack of adequate occupational information on their records.

Questionnaires were sent to a random sample of 300 women in Class VII, examined in the second half of 1974. Social-class comparisons between the originally unclassified and the remaining 6 classes were based on the results of the questionnaires for the former, and analyses of screening over the 5-year period 1971–1976 for the latter. For comparisons of class VII with the remaining classes on the basis of other characteristics (age and marital status in particular) screening data were used which included all women in the programme up to June 1976.

There were 235 codable replies (78%). Distribution of these 235 women by social class showed only small and insignificant differences from the distribution of all other women screened from 1971 to 1976 (Table I). It also showed that the marked under-representation of women in the lower classes, IV and V, was not altered by the redistribution of women formerly unclassified. The distribution of the sample was also used to reallocate all previously unclassified who had had abnormal smears. The estimates of abnormal smear rates calculated from these figures show that a strong social-class gradient is still evident (Table I).

Comparisons of all screened women in Class VII with those in the remaining 6 classes (1965–1976), however, revealed large and statistically significant differences in distribution by marital status and age. Those in Class VII include a significantly greater proportion of widows and divorcees than does the remainder of the screened population (15% versus 3·1%: $P < 0{\cdot}001$, Table II). They include much greater proportions of the elderly (6·9% were over 60 years old compared with 2% in the rest of the screened population) and those aged under 25 (40% compared with 18·4%, respectively).

The lack of any significant difference in class composition between the sample of women in Class VII and the remaining screened population indicates that a social-class factor is unlikely to be responsible for the high rate of abnormal findings among women who could not previously be classified. In fact, the explanation for the very high rate of abnormal smears in this group can be accounted for by the very characteristics that in many cases make classification difficult.

Both widows and, particularly, divorcees are known to be at high risk of having abnormal cytological smears (Sansom *et al.*, Sibary *et al.*, 1977). These same women are also, by virtue of their marital status, more likely to consider questions put to them concerning their husband's occupation inappropriate, and

TABLE I.—*Social-class Distribution*

| Social class | Sample of previously unclassified Class VII No. | % | Screened women 1971–76 No. | % | N. W. Region* population % | Abnormal smear rates per 1000 women 1965–76 No. | Rate | Re-estimated abnormal smear rates per 1000 women, 1965–76 No. | Rate |
|---|---|---|---|---|---|---|---|---|---|
| I | 12 | 5·1 | 16,025 | 5·2 | 4·9 | 137 | 4·66 | 164 | 4·96 |
| II | 45 | 19·1 | 63,834 | 20·6 | 18·5 | 730 | 6·92 | 871 | 7·31 |
| III | 117 | 49·8 | 166,268 | 53·8 | 50·5 | 2,900 | 9·63 | 3,452 | 10·25 |
| IV | 41 | 17·4 | 46,518 | 15·0 | 17·7 | 1,024 | 12·88 | 1,218 | 13·24 |
| V | 18 | 7·7 | 14,989 | 4·9 | 8·2 | 478 | 17·16 | 568 | 17·02 |
| VI | 2 | 0·9 | 1,645 | 0·5 | 0·2 | 31 | 10·15 | 38 | 10·27 |
| VII | | | (51,648) | | | 1,011 | 14·12 | | |
| Total | 235 | 100·0 | 309,279 (360,927) | 100·0 | 100·0 | 6,311 | 10·21 | 6,311 | 10·21 |

* 1971 Sample Census.

TABLE II.—*Distribution by Marital Status* (%)

| Marital state | Unclassifiable, Class VII (n = 71,610) | Other women, Classes I-VI (n = 546,321) |
|---|---|---|
| Single | 30·6 | 10·6 |
| Married | 54·4 | 86·3 |
| Widowed and divorced | 15·0 | 3·1 |
| (Widowed) | (8·7) | (1·9) |
| (Divorced) | (6·3) | (1·2) |
| Total | 100·0 | 100·0 |

hence to offer responses which are subsequently uncodable (e.g., "no longer married", "husband deceased"). Older women, another well-established group at higher risk and one more abundant among the unclassified, are also more likely to be allocated to Class VII by answering that they or their husbands are "retired".

Despite their high rate of abnormal smears, the redistribution of women in Class VII had little effect on the class gradient in risk of the disease. The rates for those in the lowest social classes are confirmed as the highest, and are perhaps even underestimated in that the distribution of unclassified women with normal smears, applied to those with abnormal smears, has probably yielded fewer than the real numbers in Classes IV and V.

The excess of abnormal smears in Class VII appears to be determined mainly by the large proportion of widowed/divorced and elderly women. Thus, although the redistribution of women who were originally unclassified does not significantly modify the under-representation of those from the lower social classes, it provides valuable confirmation of the strong social-class gradient found in previous screening analyses which had, for lack of certain information, to be based on the exclusion of this 10–15% of the programme's population.

### REFERENCES

REGISTRAR-GENERAL (1961) The Registrar-General's *Decennial Supplement: England and Wales, Occupational Mortality Tables.* HMSO.

SANSOM, C. D., WAKEFIELD, J. & YULE, R. (1971) Cervical cytology in the Manchester area: changing patterns of response. *Med. Officer*, **123**, 357.

SIBARY, K., DAVIS, F., WAKEFIELD, J. & YULE, R. (1977) Women with cervical cancer detected through population screening: implications for health education. *Int. J. Health Education*, **20**, 205.

WAKEFIELD, J. (Editor) (1972) *Seek Wisely to Prevent.* London: HMSO.

WAKEFIELD, J. & SANSOM, C. D. (1966) Profile of a population of women who have undergone a cervical smear examination. *Med. Officer*, **116**, 145.

WAKEFIELD, J., YULE, R., SMITH, A. & ADELSTEIN, A. (1973) Relation of abnormal cytological smears and carcinoma of cervix uteri to husband's occupation. *Br. Med. J.*, **2**, 142.


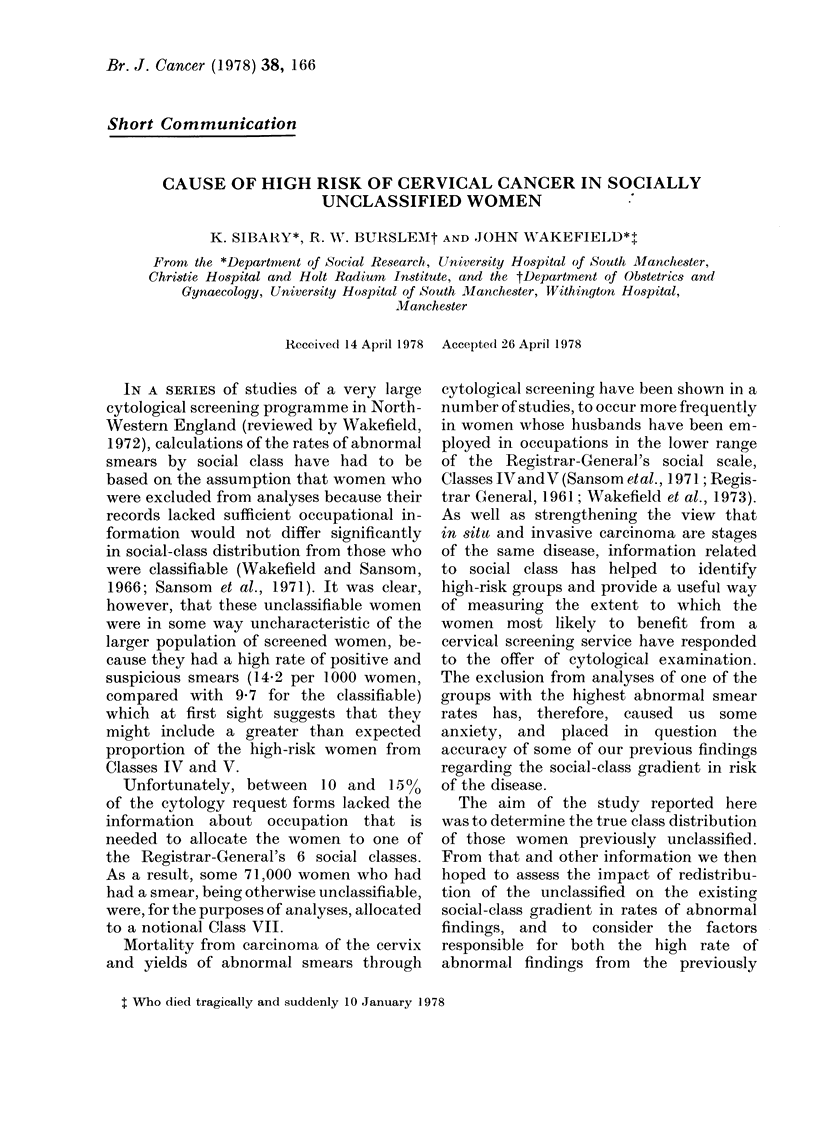

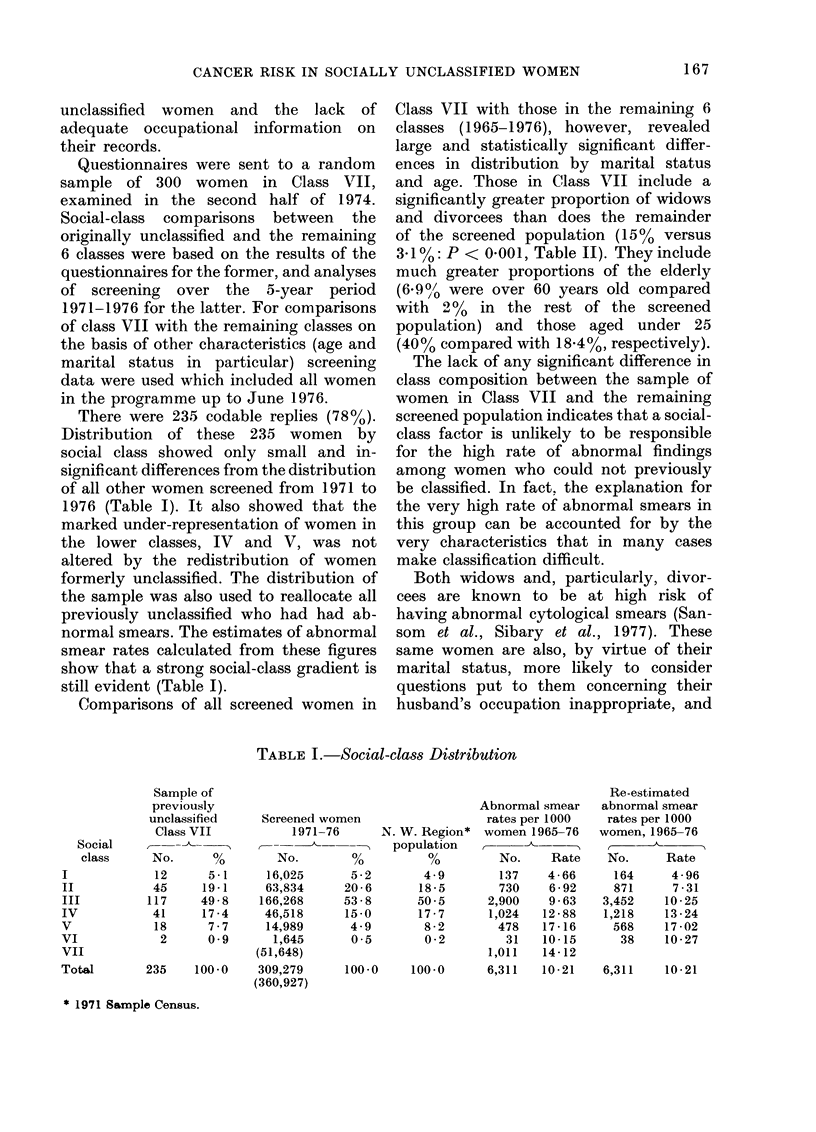

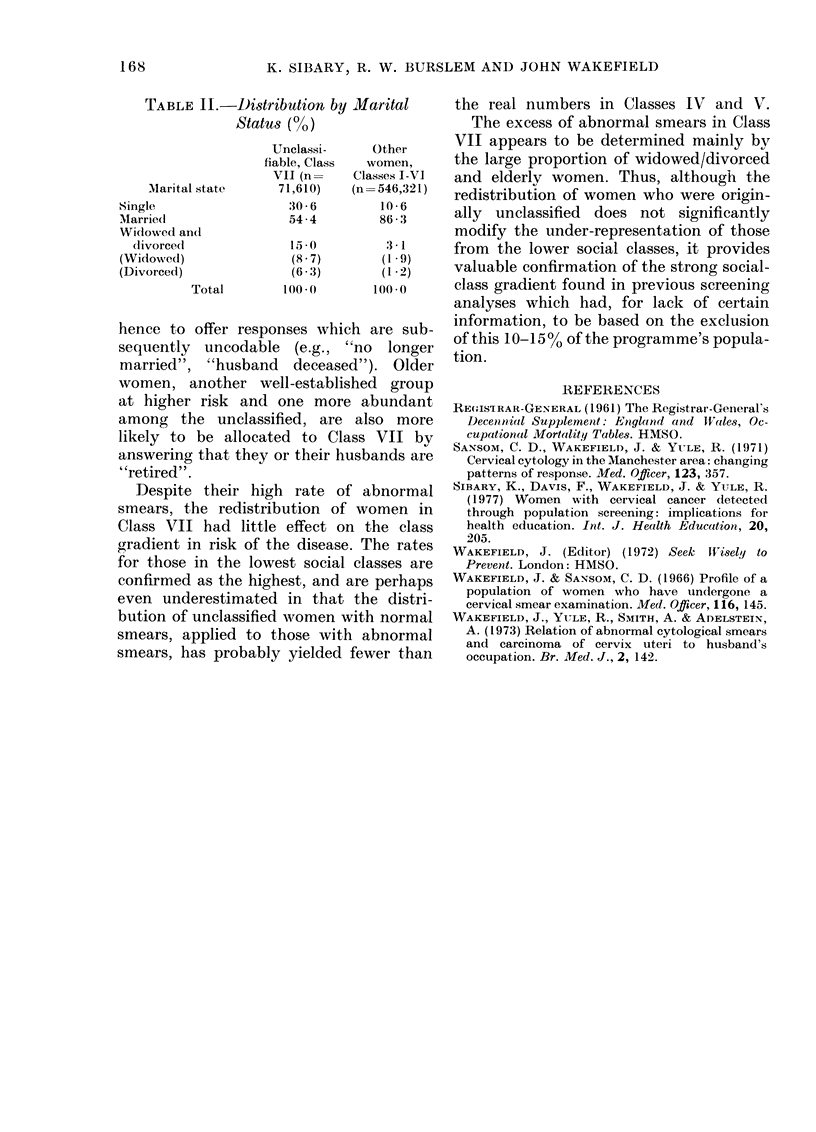


## References

[OCR_00341] Sibary K., Davis F., Wakefield J., Yule R. (1977). Women with cervical cancer detected through population screening: implications for health education.. Int J Health Educ.

[OCR_00356] Wakefield J., Yule R., Smith A., Adelstein A. M. (1973). Relation of abnormal cytological smears and carcinoma of cervix uteri to husband's occupation.. Br Med J.

